# P-1428. Real-World Effectiveness of 20-Valent Pneumococcal Conjugate Vaccine among Adults 65-74, 75-84, and ≥85 Years of Age in the United States

**DOI:** 10.1093/ofid/ofaf695.1615

**Published:** 2026-01-11

**Authors:** Amanda C Miles, Lindsay Grant, Jelena Vojicic, Jeffrey T Vietri, Summer rosenstock, Huihua Li, Alison E Randall, Xin Zhao, Wencheng Zhu, Bobby Zhao, Anan Zhou, Christian Theilacker, Jennifer Moisi, Luis Jodar, Paul Palmer, Alejandro D Cane, Paula Peyrani

**Affiliations:** Pfizer, New York, NY; Pfizer Inc., Collegeville, PA; Pfizer Canada, Kirkland, QC, Canada; Pfizer, Inc., Collegeville, Pennsylvania; Pfizer Inc, Collegeville, Pennsylvania; Pfizer, Inc, Collegeville, Pennsylvania; Pfizer, Inc., Collegeville, Pennsylvania; Genesis Research Group, Hoboken, New Jersey; Genesis Research Group, Hoboken, New Jersey; Genesis Research Group, Hoboken, New Jersey; Genesis Research Group, Hoboken, New Jersey; Pfizer Inc., Collegeville, PA; Pfizer Vaccines, Collegeville, Pennsylvania; Pfizer Vaccines, Collegeville, Pennsylvania; Pfizer Vaccine Medical Development, Scientific & Clinical Affairs , Collegeville PA, Collegeville, PA; Pfizer, New York, NY; Pfizer, Inc, Collegeville, Pennsylvania

## Abstract

**Background:**

The 20-valent pneumococcal conjugate vaccine (PCV20) was licensed by the FDA in 2021 for the prevention of vaccine type (VT) invasive pneumococcal disease (IPD) and pneumococcal pneumonia based on immunologic non-inferiority criteria. No clinical efficacy or vaccine effectiveness (VE) data has been reported for PCV20. The aim of this study was to evaluate age-group PCV20 VE against all IPD and all-cause pneumonia (ACP) among older adults in the US.

**Figure:**
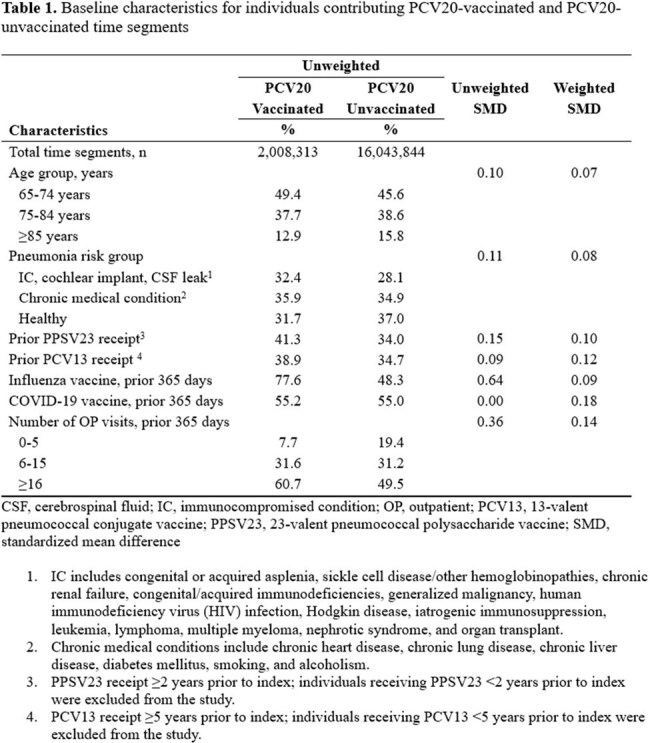

**Figure:**
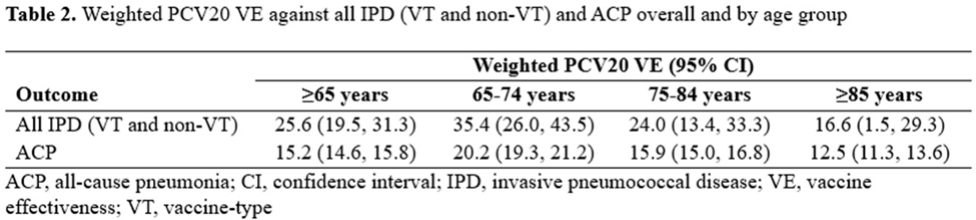

**Methods:**

In this retrospective time-segment design, individuals age ≥65 years were followed through the Medicare database from July 2022 to June 2024 for the first episode of all IPD (VT and non-VT) and ACP based on ICD-10-CM coding. We compared PCV20 vaccinated to unvaccinated adults with Medicare insurance for ≥1 year prior, excluding those who received 23-valent pneumococcal polysaccharide vaccine (PPSV23) < 2 years, PCV13 < 5 years, PCV20, or PCV15 prior to the start of study follow-up. The cohort was stratified by age group (65-74, 75-84, ≥85 years). Inverse probability weighting was used to control for confounding and dropout. Cox models estimated hazard ratios (HR) and VE was calculated as (1 – HR) x 100%. The weighted vaccine preventable disease incidence rate (IR)(VPDI) per 100K person-years (PY) was calculated as (IR_unvaccinated_ – IR_vaccinated_).

**Figure:**
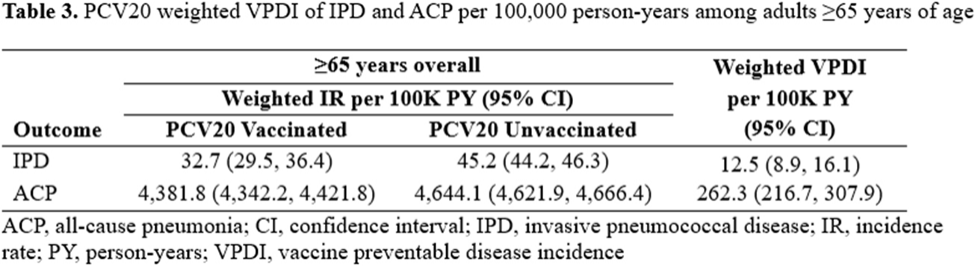

**Results:**

16.5 million adults were included, of whom 12.2% received PCV20 during follow-up. After weighting and compared to individuals with PCV20-unvaccinated segments, a higher proportion of individuals contributing vaccinated time had >5 outpatient visits in the prior year and had received a PPSV23, PCV13, or COVID-19 vaccine (Table 1). PCV20 VE against all IPD was 25.6% among adults ≥65 years of age overall and 35.4%, 24.0%, and 16.6% among adults 65-74, 75-84, and ≥85 years of age, respectively (Table 2). PCV20 VE against ACP was 15.2% among adults ≥65 years of age overall and 20.2%, 15.9%, and 12.5% among adults 65-74, 75-84, and ≥85 years of age, respectively (Table 2). Among adults ≥65 years of age overall, PCV20 vaccination prevented 12.5 cases of IPD and 262.0 cases of ACP per 100K PY (Table 3).

**Conclusion:**

This study presents the first real-world age-group specific estimates of PCV20 VE and confirms PCV20 effectiveness against IPD and ACP among adults 65-74, 75-84, and ≥85 years of age.

**Disclosures:**

Amanda C. Miles, MPH, Pfizer: Employee of Pfizer Inc.|Pfizer: Stocks/Bonds (Public Company) Lindsay Grant, PhD, MPH, Pfizer: Employee|Pfizer: Stocks/Bonds (Private Company) Jelena Vojicic, MD, Pfizer Inc.: Employee|Pfizer Inc.: Stocks/Bonds (Public Company)|Pfizer Inc.: Stocks/Bonds (Public Company) Jeffrey T. Vietri, PhD, Pfizer Inc: Employment|Pfizer Inc: Stocks/Bonds (Public Company) Summer rosenstock, PhD, MHS, Pfizer: Stocks/Bonds (Public Company) Huihua Li, MS, MD, Pfizer Pharmaceutical: employee|Pfizer Pharmaceutical: Stocks/Bonds (Public Company) Alison E. Randall, MPH, PMP, Pfizer: Employment|Pfizer: Stocks/Bonds (Public Company) Bobby Zhao, PhD, Pfizer: Advisor/Consultant|Pfizer: Employee of Genesis Research Group, which has received consulting fees from Pfizer Inc. Anan Zhou, MPH, Pfizer Inc.: Employee of Genesis Research Group, which has received consulting fees from Pfizer Inc. Christian Theilacker, MD, DTM&H, Pfizer Inc: Stocks/Bonds (Public Company)|Pfizer Inc: Stocks/Bonds (Public Company) Jennifer Moisi, PhD, Pfizer Vaccines: Employer|Pfizer Vaccines: Stocks/Bonds (Public Company) Luis Jodar, PhD, Pfizer Inc: Stocks/Bonds (Public Company) Paul Palmer, PhD, Pfizer Inc: Employee|Pfizer Inc: Stocks/Bonds (Public Company) Alejandro D. Cane, MD, PhD, Pfizer Inc.: All authors are employees of Pfizer Inc. and may hold stock and/or stock options of Pfizer Inc. Paula Peyrani, MD, Pfizer, Inc: Employee|Pfizer, Inc: Stocks/Bonds (Public Company)

